# Some Remarks upon a Quotation from the Medical and Physical Journal for June 1818, Page 499, Relating to Dr. Armstrong's Treatise on Scarlatina, &c.

**Published:** 1818-12

**Authors:** T. Parkinson


*For the London Medical and Physical Journal.*

*Some Remarks upon a Quotation from the Medical and Physical Journal for June* 1818, *page* 499, *relating to Dr. Armstrong's Treatise on Scarlatina, &c.*; by T. Parkinson, M.D.

"SCARLET Fever is divided" (by Dr. Armstrong), "as by Dr. Willan, into Simplex, Anginosa, and Maligna. We mention this, not to undervalue Dr. Armstrong, because we conceive a better could not be made."

Remark.—Such distinctions are unnecessary and confusing; and they, moreover, lead to erroneous practical deductions.

All contagious diseases, as they are called, are *enyphantitis*,—that is, inflammation of membrane, varying in their characters according to the nature of the virus by which they are respectively produced; not only in their first appearances, but also in their progress and termination, each individual disease generating its identical virus.

In scarlatina simplex, anginosa, and maligna, there is no real distinction of character: true, there is a manifest difference in the symptoms, which depend solely upon the degree of intensity of scarlatina. They are, therefore, only varied types of an individual disease, malignant in all its forms; and it is of no small importance to remark, that the virus generated in the progress of Dr. Armstrong's scarlatina simplex will produce in a second person, perhaps, his scarlatina anginosa, and in a third his scarlatina maligna.

Scarlatina simplex is intended to denote the lowest intensity: it is nevertheless scarlatina, and may prove as prolific a parent of scarlatina anginosa, or of scarlatina maligna, as either of the two last divisions. And on the same grounds it is urged, that either scarlatina anginosa or scarlatina maligna may prove the progenitor of scarlatina simplex only.

How then can distinctions of an individual disease be admitted into a system of nosology? Let it be remembered, that all Dr. Armstrong's divisions afford equally protection against a future attack of scarlatina.

Distinguishing terms must be limited to such as are expressive of identities of diseases, and cannot extend beyond classes and genera. Such terms may, however, be either *cletic*, governed by the anatomical table, denoting the precise nature of the affection, and the peculiar genus of solid diseased; or *phaneric*, governed by the physiological table, denoting the peculiarity of function impaired or deranged.

Let us enquire how this disposition will be true to itself in the disease before us.

Scarlatina—inflammation of membrane, caused by the application of a peculiar animal poison. The distinguishing characteristic of membrane is intertexed fibres. ENYPHANTA, from ενυφαινω, *intexo*, to intertex or interweave. Enyphanta is fully representative of membrane, because there is no other animal solid that is entitled to the appellation, or indeed to which the term applies. By affixing the termination ITIS, signifying excess of animal or vital action, we express the precise nature of the affection,—that is, excess of vital or animal action above the limits of health in a membrane—ENYPHANTITIS. The typical adjective *remittens*, or that of *progressiva*, will detect every distinction necessary to be regarded, and at the same time will pronounce the prognosis.

The complete analysis of scarlatina, then, is given by Enyphantitis scarlatina remittens: inflammation of membrane from the scarlatina virus, remitting: Dr. Armstrong's first division, Scarlatina simplex, and of the lowest degree of intensity; prognostic favourable; fever* typhus mit. Or,

* I have given it as my opinion, that the term Fever, being retained in medical language, has been the silent cause of more misery and mortality than the plague or the sword; yet I have felt myself compelled, in this instance, to make use of the same term, to avoid being misunderstood: in future, I shall adopt HYPERBIOSIS, from Υπερ βιωσις—*super actio vivendi:* excess of exercise, above the limits of health; including all inflammatory diseases.

Enyphantitis scarlatina progressiva: inflammation of membrane from scarlatina virus, progressive; proceeding progressively to gangrene and death. Fever typhus grav.; prognostic unfavourable: Dr. Armstrong's second and third divisions of scarlatina.

But, if it be wished to distinguish the disease by its phanerics, or by evidences which arise out of diseased functions, it will be necessary to be governed by the physiological table.

The function of membrane is interposition, Parenthesis. The exercise of this function is excessive above the limits of health, in some distinguishable part of the body, under scarlatina, and will be fully expressed, both as to diagnosis and prognosis, by Parenthitis scarlatina remittens, or progressiva.

It is worthy of remark, that scarlatina simplex of Dr. Armstrong will always be remittent, on account of the slightness of its intensity, and the constitutional affections, called fever, will correspond in remissions: but the moment it is progressive, it is Dr. Armstrong's scarlatina anginosa, or his scarlatina maligna; and the constitutional sufferings, called fever, will correspond in progression towards death.

---

